# Lessons learned: Chronic idiopathic constipation patient experiences with over-the-counter medications

**DOI:** 10.1371/journal.pone.0243318

**Published:** 2021-01-11

**Authors:** Brian E. Lacy, Elizabeth P. Shea, Machelle Manuel, Jessica L. Abel, Hancheng Jiang, Douglas C. A. Taylor

**Affiliations:** 1 Gastroenterology and Hepatology, Mayo Clinic, Jacksonville, FL, United States of America; 2 Medical Writing and Publications, Ironwood Pharmaceuticals, Inc., Boston, MA, United States of America; 3 Ironwood Pharmaceuticals, Inc., Boston, MA, United States of America; 4 Global Health Economics & Outcomes Research, AbbVie Inc., Madison, NJ, United States of America; 5 Health Economics & Outcomes Research, Ironwood Pharmaceuticals, Inc., Boston, MA, United States of America; The University of North Carolina at Chapel Hill, UNITED STATES

## Abstract

**Introduction:**

Chronic idiopathic constipation (CIC) is a prevalent functional gastrointestinal disorder diagnosed based on patient-reported symptoms and the absence of structural gastrointestinal abnormalities. Individuals with CIC typically institute dietary changes and use stool softeners or over-the-counter (OTC) laxatives, possibly at the direction of a healthcare provider, before prescription medications for CIC are initiated. Although highly prevalent, there is limited information regarding CIC patient experiences with OTC medications.

**Methods:**

This post-hoc analysis used patient-reported data from a questionnaire administered during patient screening for a prospective linaclotide Phase 3b clinical trial in patients with CIC (N = 1482 screened). The questionnaire asked patients to report their experiences with OTC CIC medications over the preceding 6 months.

**Results:**

Among patients with screening responses (N = 1423), most were female (85%) and white (66%), with a mean age of 48.9 years. A high proportion of patients had used one or more OTC medications (70% had ≥1 OTC; 19% had ≥3 OTCs), with the majority being bisacodyl (33%) and polyethylene glycol (30%). The most commonly cited reason for stopping an OTC medication was insufficient symptom relief (17–40%). The majority of patients taking OTC medications reported no or little satisfaction with the medication’s effect on their constipation (62%) and CIC-specific abdominal symptoms (78%). Many patients had little to no confidence in bowel movement (BM) frequency after taking OTC medications and their confidence in their ability to predict BM timing was also low (49–81% not at all confident).

**Conclusions:**

Treatment effects on individual CIC symptoms, predictability of bowel habits, and satisfaction with treatment are all important factors for healthcare providers and patients to consider when establishing an effective treatment regimen for CIC.

**Trial registration number:**

NCT01642914

## Introduction

Chronic idiopathic constipation (CIC) is a symptom-based functional gastrointestinal disorder with an estimated prevalence ranging from 12% to 19% among adults in North America [1]. No definitive biomarker exists for identifying CIC and thus the diagnosis is based on patient-reported bowel signs and symptoms in the absence of structural gastrointestinal abnormalities. The Rome IV diagnostic criteria for functional constipation represent the current gold standard for the diagnosis of CIC and include thresholds for infrequent bowel movements (BMs), straining during defecation, hard/lumpy stools, a sensation of incomplete evacuation, a sensation of anorectal obstruction/blockage, and the need for manual manipulations [2]. In addition to bowel symptoms, many patients with CIC also experience abdominal symptoms such as pain, bloating, discomfort, gassiness, fullness, and stomach cramps [1, 3, 4]. CIC has a significant negative impact on patients’ health-related quality of life [5, 6]. Among the factors contributing to diminished health-related quality of life and disease burden are the worry and uncertainty about symptom onset, the persistence of symptoms, poor response to over-the-counter (OTC) medications, and the unpredictable timing of BMs in response to medical therapy [5, 7].

Individuals with symptoms of CIC typically make self-guided dietary changes and use stool softeners or OTC laxatives, possibly at the direction of a healthcare provider, before prescription medications for CIC are initiated. Several types of OTC laxatives are commonly used, including osmotic agents (polyethylene glycol [PEG] and lactulose), bulk-forming agents (psyllium husk and wheat dextrin), and stimulants (senna and bisacodyl) [8, 9].

In a survey of patients with CIC (N = 1223), 40% reported using an OTC laxative and had tried an average of three OTC products before consulting with their healthcare provider [10]. A survey of US gastroenterologists (N = 830) indicated that approximately 97% initially recommend OTC medications for chronic constipation rather than a prescription medication [11]. Patients who do not respond to OTC medications may seek the assistance of a healthcare provider who can initiate therapy with a prescription medication. Several prescription medications are now available for the treatment of adults with CIC, including linaclotide, lubiprostone, plecanatide, and lactulose. Linaclotide, lubiprostone, and plecanatide are approved specifically for CIC and lactulose is approved for chronic constipation by the US Food and Drug Administration and for CIC by other regulatory agencies.

Despite the high prevalence of CIC among US adults and its significant impact on quality of life and costs, there is limited information regarding patient experiences with medications used to treat CIC [12]. Patient-reported data would provide valuable information to healthcare providers that would help them guide patient treatment. The goal of this analysis was to use patient-reported questionnaire data from screening for a prospective Phase 3b clinical trial to help understand the experiences of patients with CIC in using OTC medications over the preceding 6 months.

## Methods

### Study design

This post-hoc analysis used data from LIN-MD-04, a Phase 3b, 12-week, multicenter, randomized, double-blind, placebo-controlled, parallel-group study (NCT01642914). The study aimed to assess the efficacy and safety of linaclotide at doses of 145 μg and 290 μg administered once daily in patients with CIC with prominent abdominal symptoms. Patients were screened at 141 clinical sites (136 in the United States and five in Canada) and the trial was conducted from August 2012 to May 2013. Full details of the trial methodology and primary results have been reported previously by Lacy et al [13]. The trial was designed, conducted, and reported in compliance with the ethical principles that have their origins in the Declaration of Helsinki and the principles of Good Clinical Practice guidelines. Institutional Review Boards approved the protocol and all trial procedures for all trial centers (Quorum Review Institutional Review Board, Western Institutional Review Board, Mercy Medical Center Institutional Review Board, University of Oklahoma Health Sciences Center Institutional Review Board). All patients gave written informed consent prior to participating in any trial-related procedures.

### Patient population

The study population comprised adult patients who met the modified Rome II criteria for chronic constipation upon entry [14]. Additionally, eligible patients reported an average of ≤6 spontaneous BMs and <3 complete spontaneous BMs, with an average abdominal bloating score of ≥5 (0–10-point numerical rating scale), during the 14-day baseline period [15]. Patients with prior exposure to linaclotide were excluded from the trial. Prior exposure to lubiprostone and lactulose was permitted; plecanatide was not available at the time this study was conducted.

### Prior medication assessments

At study screening, patients were asked to complete a Prior Medication Questionnaire (PMQ), assessing their experience over the previous 6 months with select OTC medications used to treat their CIC symptoms. Medications included bisacodyl, docusate sodium, PEG, psyllium husk, senna, and wheat dextrin (both trade and generic names were provided). Specific details of each prior therapy were recorded (how often it was used [daily, as needed, other], duration of use, and, if applicable, the reason(s) for discontinuation [including side effects and costs]).

Patients also reported their experiences in relation to the following:

- Satisfaction with the medication’s ability to relieve constipation, abdominal pain, and abdominal bloating, each assessed on a 5-point scale (1 = not at all satisfied; 2 = a little satisfied; 3 = moderately satisfied; 4 = quite satisfied; 5 = very satisfied)
- Confidence in having ≥1 BM every other day, assessed on a 3-point scale (1 = not at all confident; 2 = somewhat confident; 3 = very confident)
- Ability to predict timing of BMs after medication use, assessed on a 3-point scale (1 = not at all confident; 2 = somewhat confident; 3 = very confident)

The analysis population was comprised of screened patients who completed at least one assessment on the PMQ. Descriptive statistics were used for the patient demographics and baseline symptoms. Summary statistics were used for the number of medications tried and discontinued, PMQ responses overall, and by treatment reported. Analyses were conducted in SAS version 9.4 (SAS Institute, Cary, NC).

## Results

### Demographics and prior medications

Of 1482 patients screened, 1423 (96%) provided responses to the PMQ. Patients were predominantly female (85%) and white (66%), with a mean age of 48.9 years (Table 1). Of the 1423 patients who responded to the PMQ, 994 (70%) reported trying ≥1 OTC medication for CIC during the previous 6 months, while 273 (19%) reported trying ≥3 medications (Table 2). Approximately 44% of patients had tried and stopped one or more medications and 30% had tried and stopped one or more medications due to insufficient relief of symptoms.

**Table 1:** Demographics and clinical characteristics (PMQ population).

Characteristic	Completed prior medication form
	(N = 1423)
**Sex, n (%)**	
Female	1210 (85.0)
Male	213 (15.0)
**Age, years, mean (SD)**	48.9 (14.1)
**Age group, years, n (%)**	
18–39	378 (26.6)
40–64	857 (60.2)
≥65	188 (13.2)
**Race, n (%)**	
White	945 (66.4)
Black or African American	437 (30.7)
Other	20 (1.4)
Asian	17 (1.2)
American Indian or Alaska Native	4 (0.3)
**Body mass index, kg/m**^**2**^**, mean (SD)**	29.1 (6.4)

Abbreviations: PMQ = Prior Medication Questionnaire, SD = standard deviation.

**Table 2:** Number of OTC medications patients tried during the previous 6 months (N = 1423).

Number of OTC medications	Patients who tried OTC medications, n (%)	Patients who tried and stopped OTC medications,	Patients who tried and stopped OTC medications due to insufficient symptom relief,
		n (%)	n (%)
0	429 (30.2)	798 (56.1)	997 (70.1)
1	483 (33.9)	306 (21.5)	236 (16.6)
2	238 (16.7)	139 (9.8)	103 (7.2)
3	148 (10.4)	96 (6.8)	57 (4.0)
>3	125 (8.8)	84 (5.9)	30 (2.1)

Percentages are based on the full survey population (i.e., all patients who responded to the survey, regardless of prior medication use). Abbreviation: OTC = over-the-counter.

The usage and duration of use of OTC medications are summarized by treatment in Table 3. The OTC medications used by the most patients were bisacodyl (33%) and PEG (30%); 11% of those who used bisacodyl used them daily, as did 44% of those who used PEG. Daily use was reported by 26–52% of the patients who used other OTC medications. Up to 40% of patients had been using an OTC medication for more than 1 year. Reasons for discontinuing are summarized by treatment in Table 3. Across medications, insufficient relief of bowel symptoms was the most commonly cited reason (17–40%) for treatment discontinuation, followed by insufficient relief of abdominal symptoms (9–23%), side effects (6–13%), and cost (2–4%). Wheat dextrin and bulk agents were the most frequently discontinued treatments.

**Table 3:** Medication use and stoppage (N = 1423).

	PEG	Bulk	Wheat dextrin	Bisacodyl	Senna	Stool softeners
**Medication used in previous 6 months, n (%)**	423 (30)	322 (23)	135 (9)	463 (33)	290 (20)	337 (24)
**How do (or did) you use this medication?, n (%)**						
Daily	184 (44)	167 (52)	64 (47)	50 (11)	74 (26)	126 (38)
As needed (PRN)	220 (52)	134 (42)	64 (47)	384 (83)	189 (65)	197 (59)
Other	19 (5)	20 (6)	7 (5)	29 (6)	27 (9)	11 (3)
**How long have you taken (or did you take) this medication?, n (%)**						
<1 week	49 (12)	37 (12)	23 (17)	57 (12)	40 (14)	29 (9)
>1 week but <1 month	84 (20)	89 (28)	40 (30)	72 (16)	52 (18)	52 (16)
>1 month but <3 months	64 (15)	44 (14)	25 (19)	42 (9)	30 (10)	54 (16)
>3 months but <6 months	48 (11)	32 (10)	12 (9)	43 (9)	28 (10)	33 (10)
>6 months but <1 year	55 (13)	44 (14)	11 (8)	65 (14)	31 (11)	46 (14)
*≥*1 year	123 (29)	75 (23)	23 (17)	184 (40)	109 (38)	120 (36)
**If you stopped taking this medication, what was the reason?, n (%)**^**a**^						
Not applicable, I am currently taking this medication	161 (34)	81 (21)	18 (11)	220 (44)	104 (33)	138 (37)
It did not improve my abdominal symptoms	66 (14)	71 (19)	38 (23)	43 (9)	42 (13)	50 (13)
It did not improve my bowel symptoms	127 (27)	129 (34)	65 (40)	84 (17)	67 (21)	121 (32)
I experienced side effects	39 (8)	42 (11)	13 (8)	65 (13)	34 (11)	22 (6)
It costs too much	16 (3)	14 (4)	7 (4)	14 (3)	8 (3)	9 (2)
Other	62 (13)	44 (12)	23 (14)	75 (15)	65 (20)	36 (10)

^a^Patients selected all that applied. Abbreviations: PEG = polyethylene glycol, PRN = pro re nata.

### PMQ assessments

**Fig 1:**
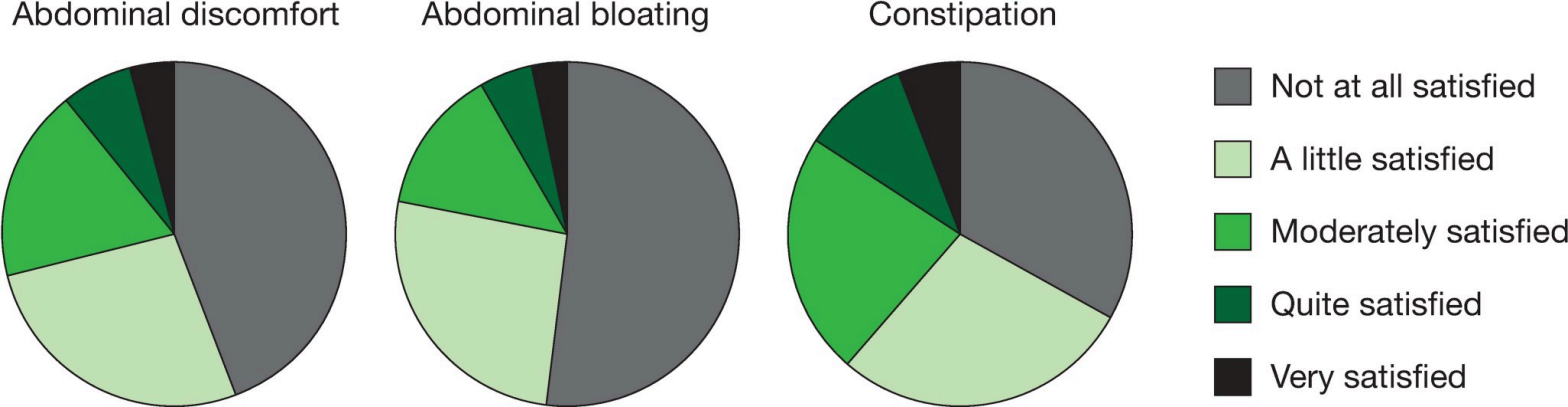
Patient satisfaction with OTC medication’s ability to relieve symptoms. Abbreviation: OTC = over-the-counter.

**Fig 2:**
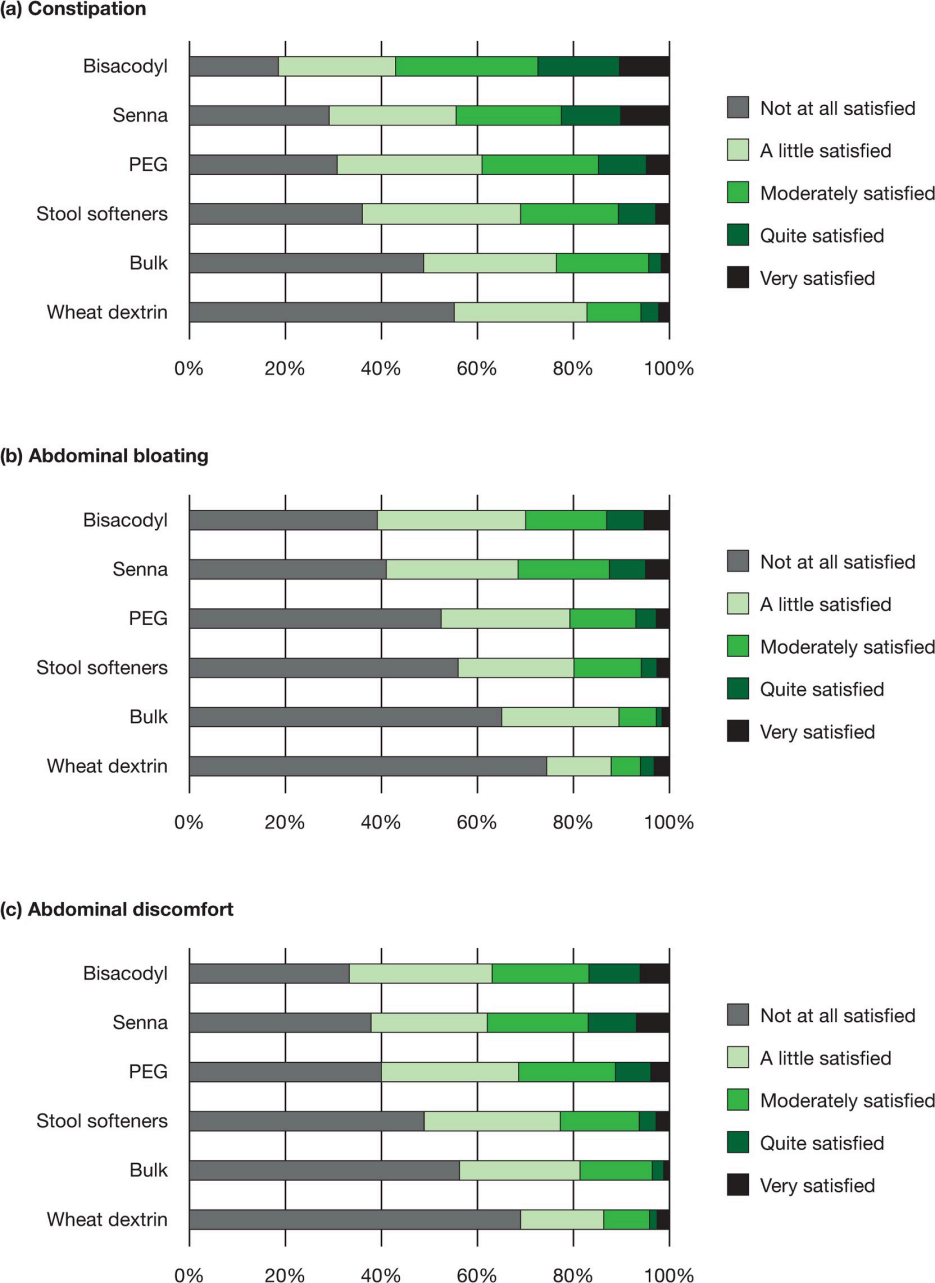
Patient satisfaction with relief of (a) constipation, (b) abdominal bloating, and (c) abdominal discomfort, by treatment. Abbreviation: PEG = polyethylene glycol.

**Fig 3:**
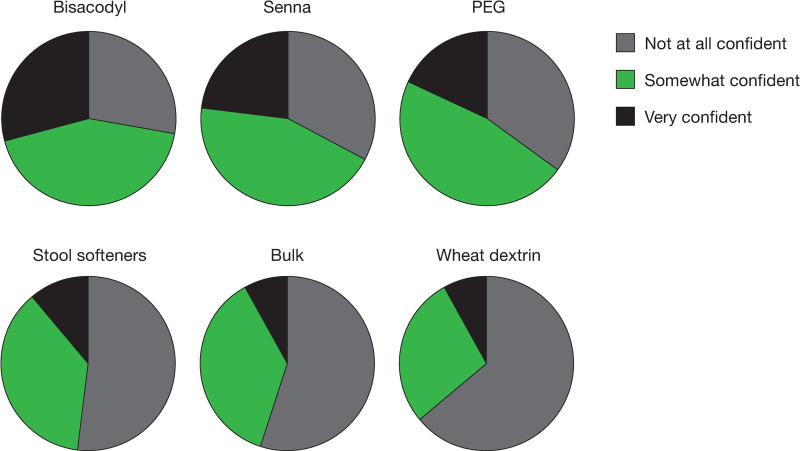
Patient confidence in having a bowel movement at least once every other day while taking an OTC medication. Abbreviations: OTC = over-the-counter, PEG = polyethylene glycol.

**Fig 4:**
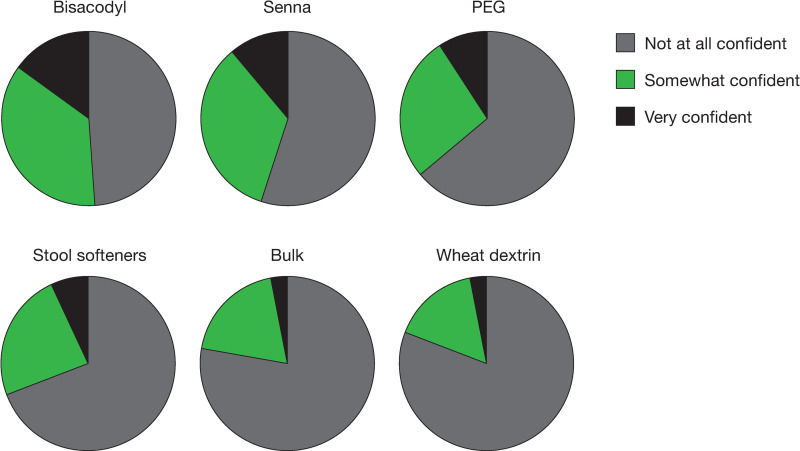
Patient confidence in predicting the timing of bowel movements after taking an OTC medication. Abbreviations: OTC = over-the-counter, PEG = polyethylene glycol.

Overall, most patients were “not at all satisfied” or only “a little satisfied” with an OTC medication’s ability to relieve the symptoms of constipation (33% and 28%, respectively), abdominal bloating (52% and 26%, respectively), and abdominal discomfort (41% and 25%, respectively) [Fig 1]. Across OTC medications, the percentage of patients who were moderately satisfied to very satisfied with the medication’s ability to relieve constipation ranged from 17% to 57%; satisfaction with the ability to relieve abdominal discomfort was 13% to 38% and satisfaction with the ability to relieve abdominal bloating was 10% to 31% (Fig 2A–2C). Few patients (8–29%) were very confident they would have a BM at least once every other day while taking an OTC medication, with the highest proportion of those who were very confident being on stimulant laxatives (Fig 3). Similarly, very few patients (3–15%) were very confident they could predict the timing of their BMs after taking an OTC medication, with 49–81% being not at all confident (Fig 4). Bisacodyl consistently had the highest levels of satisfaction among the treatments studied.

## Discussion

Research has shown patients generally begin treatment for CIC with OTC medications, and many healthcare providers also recommend these agents as first-line therapy. These agents may only provide partial relief of symptoms and patients may not be fully satisfied due to their variable efficacy and unpredictable onset of action [10]. Earlier survey-based studies have reported that patients with CIC had low levels of satisfaction with use of current OTC medications for their CIC symptoms [16–18]. Our study in a large CIC patient population shows that a high proportion of patients with CIC have tried more than one OTC medication and report stopping treatment due to insufficient symptom relief, suggesting patients may be cycling through several OTC treatments without obtaining sufficient relief of their CIC symptoms. By asking patients specific questions about experiences with OTC medications, our study results may help explain why CIC patients may be cycling through medications. Beyond general satisfaction responses, our study provides important new information and additional insights for healthcare providers by addressing specific aspects associated with OTC medications, including their ability to treat bowel and abdominal symptoms and the ability of patients to predict their BMs while taking the medications.

In our study, patient satisfaction with OTC medication appeared higher for the relief of constipation symptoms than for the relief of abdominal symptoms. Our study provided similar results to an earlier trial which showed bisacodyl effectively improved bowel function and constipation-related symptoms in patients with chronic constipation [19]. However, patient worries and concerns related to CIC symptoms and BM predictability are also important components of patient experience, treatment satisfaction, and health-related quality of life [5, 16, 17]. Patients with CIC have specifically noted being worried about not being able to move their bowels when needed and not knowing when they will be able to move their bowels [7]. This lack of predictability may have a detrimental effect on a patient’s ability to plan and participate fully in activities of daily living, including travel, work, and leisure.

Results of this study show that many patients with CIC have little to no confidence in their ability to modulate BM frequency while taking any of the OTC medication types reported, and their confidence in their ability to predict BM timing is also low. Furthermore, patient satisfaction with an OTC medication’s ability to relieve abdominal symptoms (bloating and discomfort) was lower than patient satisfaction with the medication’s ability to relieve their constipation. This is important for healthcare providers to consider, as bloating and other abdominal symptoms are common in patients with CIC and some OTC medications can even exacerbate these symptoms [3, 16, 20–22].

The present study includes the largest patient population in CIC (over 1400 patients) to report on patient-reported treatment experiences [17], adding significantly to the patient-reported evidence base on experience with OTC medications for CIC. The study shows that patients with CIC often take a number of OTC medications which have little positive impact on bowel function or other symptoms of CIC. While it is important to acknowledge the post-hoc nature of these analyses, it should be noted that questions were provided to patients at the start of the LIN-MD-04 study and all data were gathered prospectively. In addition, OTC medications for constipation may not have been represented equally and statistical analyses are limited. This was a cross-sectional survey conducted in a heterogeneous population. Given the dataset, formal statistical analyses were not appropriate and the analyses are limited to descriptive statistics. Further, the responses were based on patients’ recall over the previous 6 months, which may not have been fully accurate or complete, and the study did not take into account whether patients took multiple medications concomitantly or cycled through them one after the other. It should also be noted that this study was conducted in a subset of patients with CIC with severe bloating and may not be fully representative of the wider CIC population. As the population included in the analysis were screening patients for a Phase 3 study, there may have been some selection bias for those who did not respond well to laxatives or other OTC medications. However, this analysis does provide insight into the patient journey, i.e., some patients respond well to treatment while others cycle for a long period with limited improvement in their symptoms.

Further research is warranted to address treatment patterns over time, to provide clearer insights into patient journeys with OTC medications.

## Conclusions

CIC is a common condition with a large proportion of patients trying more than one OTC medication for relief of constipation symptoms, suggesting limited efficacy in this group of patients and the need for alternative therapies. For the majority of OTC medications, patients report no or little satisfaction with the medication’s effect on their constipation and abdominal symptoms. These are important factors for healthcare providers and patients to consider when establishing a treatment strategy for CIC. Accordingly, these results provide additional insights into OTC treatment experiences that may help guide healthcare providers to ask patients more pertinent questions about previous treatments and their effects on individual symptoms, the predictability of their bowel habits, and their satisfaction with those treatments.

## Supporting information

S1 Checklist TREND statement checklist. (PDF)
